# Reversed Midgut Rotation in an Adult With Atypical Presentation as Upper Gastrointestinal Bleeding: A Case Report

**DOI:** 10.7759/cureus.112107

**Published:** 2026-07-05

**Authors:** Sandra Rodarte-González, Marlene Abigail M Salazar Garces, David Julian Peña Hernandez, Adrián A Negreros-Osuna

**Affiliations:** 1 Radiology, Hospital Regional Instituto de Seguridad y Servicios Sociales de los Trabajadores del Estado Monterrey, Universidad de Monterrey, Monterrey, MEX

**Keywords:** anatomic malformation, body imaging ct, digestive surgery, surgery rotations, upper gastrointestinal bleed

## Abstract

Reversed midgut rotation is the rarest subtype of intestinal malrotation and is seldom encountered in adult patients.

We present the case of a 67-year-old male admitted for a six-month history of upper gastrointestinal bleeding. Contrast-enhanced abdominal computed tomography (CT) demonstrated, as an incidental finding, reversed midgut rotation, characterized by an abnormal position of the duodenum and its relationship with the mesenteric vessels and adjacent structures. Given the absence of obstructive complications, conservative, non-surgical management was deemed appropriate. Upper endoscopy revealed active bleeding, and a hemostatic clip was placed without the need for exploratory surgery. The patient showed adequate clinical response and was discharged with outpatient follow-up.

In addition to illustrating a rare case of reversed midgut rotation, this report highlights its atypical clinical and radiologic presentation and emphasizes the importance of CT in recognizing uncommon anatomic variants that may significantly influence clinical and surgical management.

## Introduction

Intestinal malrotation is an uncommon congenital anomaly resulting from a failure of the midgut to undergo its normal rotation around the axis of the superior mesenteric artery. This disrupts the normal anatomic configuration of the midgut, specifically involving the positioning of the duodenum, small intestine, and colon. While early-onset congenital cases typically present acutely in the neonatal or pediatric population, late-onset malrotation may also be identified in adults. In these late-onset cases, patients frequently have a slight female predominance [1-4]. The spectrum of these rotational anomalies includes non-rotation, incomplete rotation, and reversed rotation. Reversed midgut rotation is the rarest subtype, accounting for only 2%-4% of all cases, and is characterized by a reversed anatomical relationship that positions the transverse colon behind the superior mesenteric artery and the duodenum in front of it [2,5,6].

The abnormal fixation and positioning inherent to malrotation predispose patients to catastrophic complications, and the typical clinical presentation constitutes a surgical emergency due to duodenal obstruction, cecal volvulus, or mesenteric ischemia [3,4,7]. Nevertheless, a small number of patients remain asymptomatic or present with chronic, non-specific symptoms such as nausea, vomiting, weight loss, and recurrent abdominal pain [4,7].

Because adult patients frequently lack classic obstructive symptoms, the condition may remain undetected until it is identified as an incidental finding on routine investigations, such as endoscopic evaluations or radiographic studies performed for vague gastrointestinal complaints. We present the case of an adult patient with an atypical presentation in the form of upper gastrointestinal bleeding, aiming to emphasize the fundamental role of computed tomography (CT) in establishing the diagnosis of this entity.

## Case presentation

A 67-year-old male with a history of long-standing diabetes mellitus and congenital mitral insufficiency presented with a six-month history of generalized weakness, fatigue, and decreased appetite. Despite multiple emergency department evaluations at an outside institution, no definitive diagnosis had been established. One week before admission, he developed melena and coffee-ground emesis, accompanied by diffuse, intermittent epigastric pain, abdominal distension, nausea, and progressive weight loss. On physical examination, vital signs were stable, and the abdomen was soft, non-tender, and without peritoneal signs. At this stage, based on the chief complaint and clinical examination, the primary differential diagnoses for the upper gastrointestinal bleeding included peptic ulcer disease, erosive gastritis, and gastrointestinal malignancy. Laboratory workup was notable for a hemoglobin of 5 g/dL, prompting transfusion of two units of packed red blood cells and two units of fresh frozen plasma. Blood glucose was 94 mg/dL, consistent with adequate glycemic control in the context of his known diabetes.

**Figure 1:**
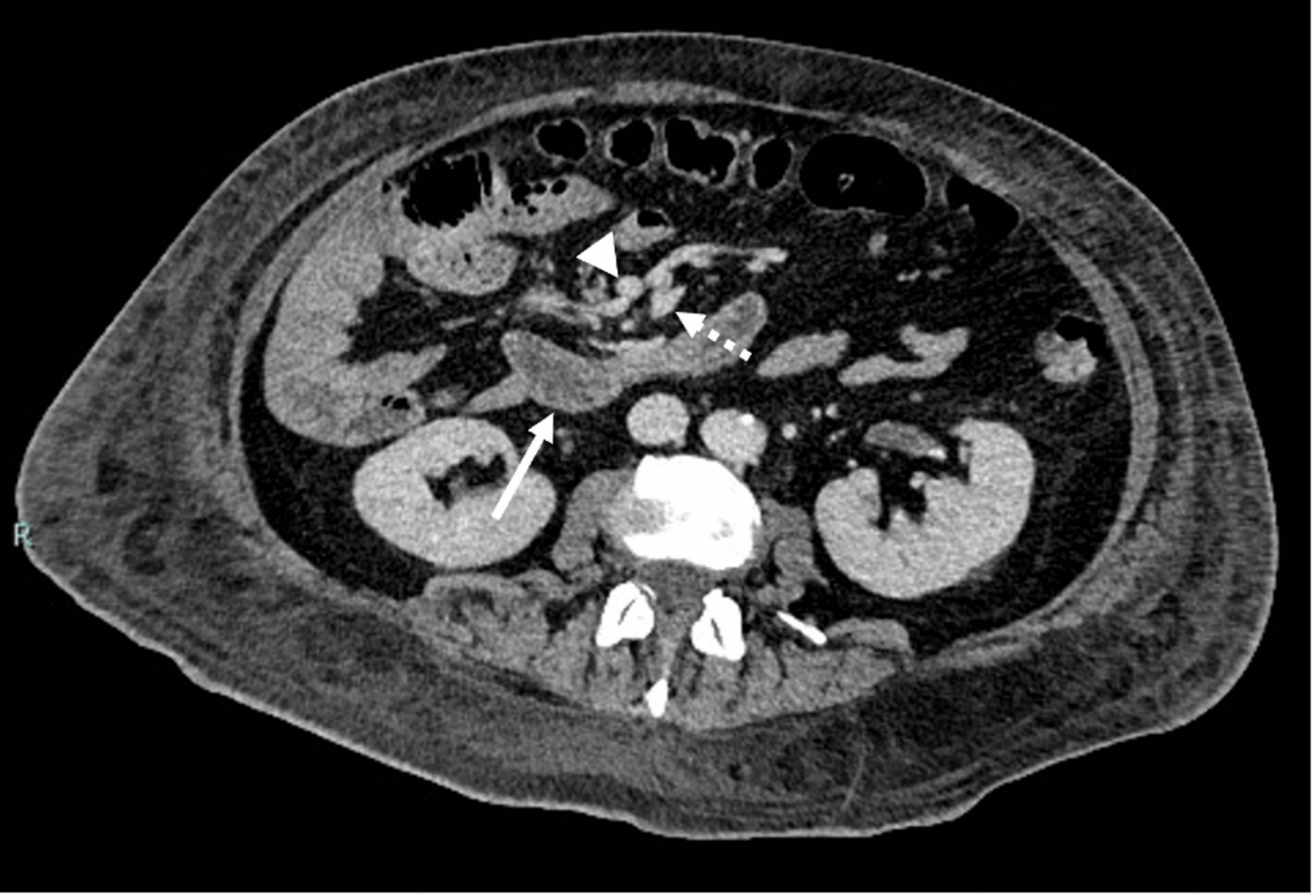
Midgut rotation Contrast-enhanced axial CT image in the venous phase demonstrating clockwise rotation of the second through fourth portions of the duodenum, compatible with reversed midgut rotation (arrow: duodenum; dashed arrow: superior mesenteric artery; arrowhead: superior mesenteric vein).

**Figure 2:**
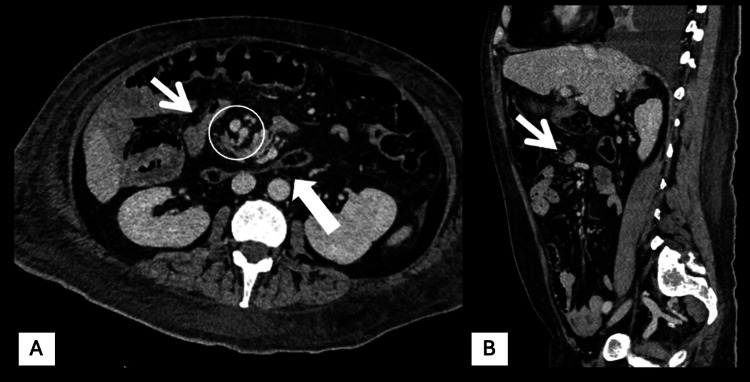
Duodenojejunal junction (A) Contrast-enhanced axial CT image in the venous phase demonstrating the duodenojejunal junction (thin arrow) located to the right of the superior mesenteric vessels (circle) (thick arrow: transverse colon). (B) Sagittal reconstruction confirming right-sided location of the duodenojejunal junction (thin arrow).

Given the non-specific symptoms and suspicion of upper gastrointestinal bleeding, contrast-enhanced CT of the abdomen was performed before endoscopy. The study demonstrated abnormal anatomy, with clockwise rotation of the second through fourth portions of the duodenum around the superior mesenteric vessels in an intraperitoneal location (Figure 1). The duodenojejunal junction was located to the right of the midline and anterior to the mesenteric vessels (Figure 2).

**Figure 3:**
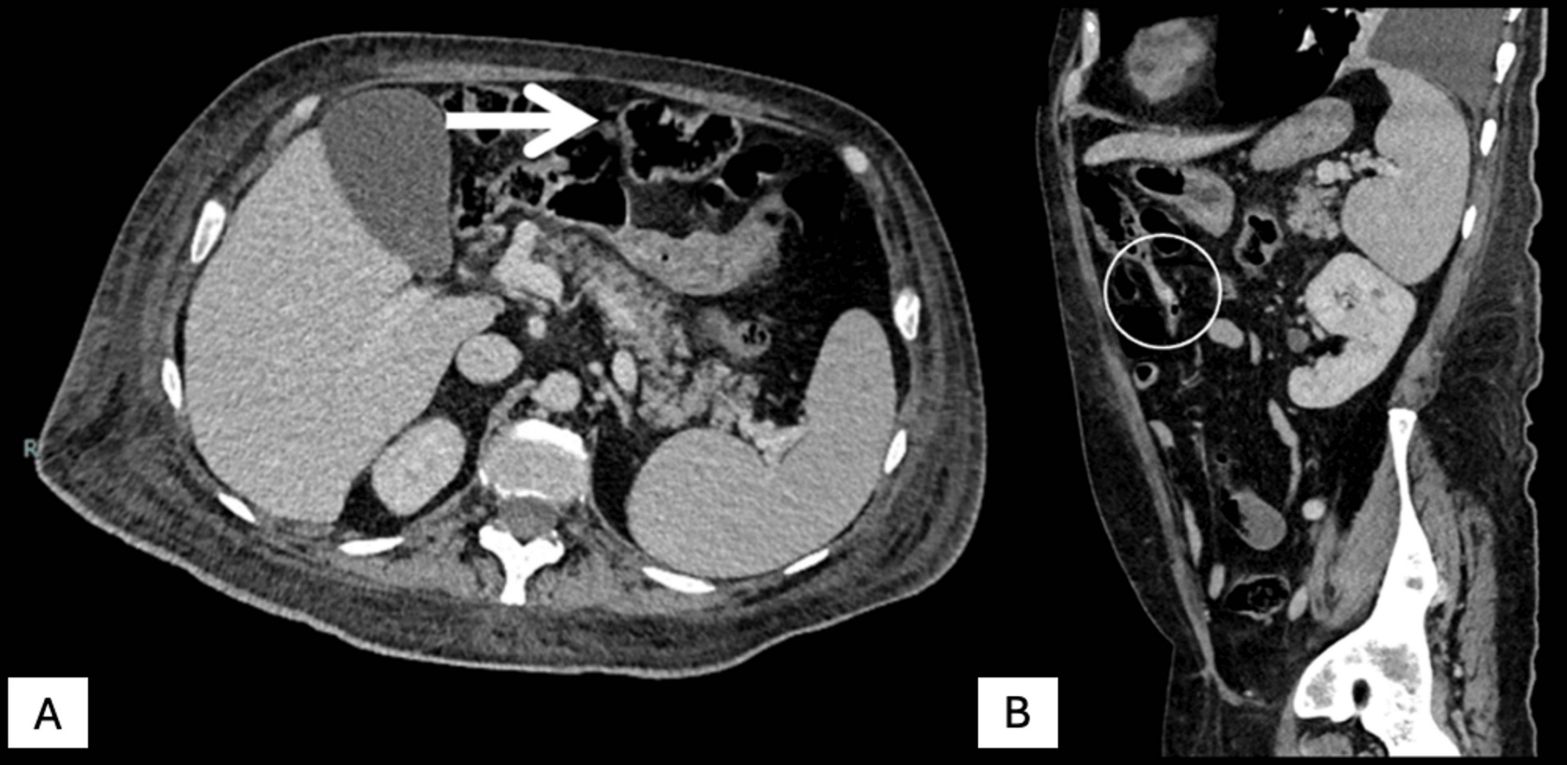
Ectopic cecum Contrast-enhanced CT images in the venous phase. (A) Axial and (B) coronal images demonstrating an ectopic cecum (white arrow) located in the left epigastrium and the appendix (white circle) in the left upper quadrant.

**Figure 4:**
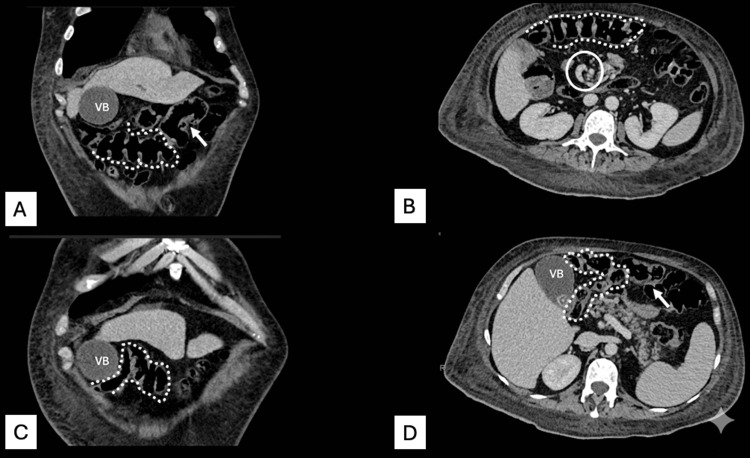
Ascending colon Contrast-enhanced CT in the venous phase demonstrating the ascending colon (dashed line) located in the epigastrium and right hypochondrium. It courses from left to right (A, B) and subsequently from anterior to posterior, medial to the gallbladder (VB) (C, D) (circle in B: mesenteric vessels; thin arrow in A and D: cecum; VB in A, C, and D: gallbladder).

**Figure 5:**
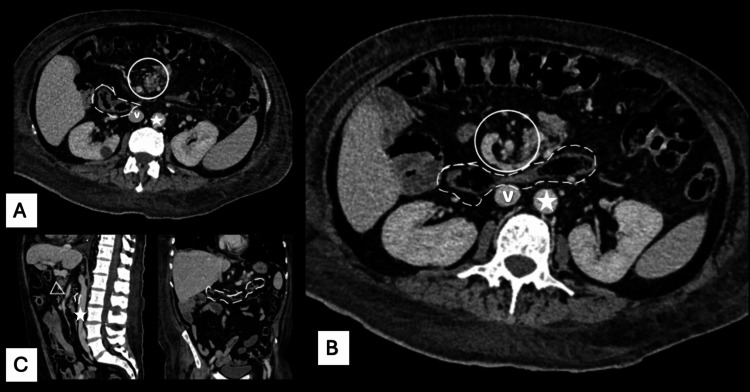
Transverse colon (A, B) Contrast-enhanced CT in the venous phase demonstrating the transverse colon (dashed line) crossing from right to left posterior to the mesenteric vessels (circle in A and B) and anterior to the inferior vena cava (V in A and B) and aorta (star in A, B, and C). (C) Sagittal and coronal reconstruction demonstrating the normal origin and course of the superior mesenteric artery (triangle) (dashed line: transverse colon; star: aorta).

The cecum appeared intraperitoneal and was in the epigastrium at the midline, with leftward displacement (Figure 3). A tubular structure compatible with the appendix was identified in the left upper quadrant, extending inferiorly and posteriorly toward the mesogastrium (Figure 3). The ascending colon was located along the midline, predominantly in the epigastrium and right hypochondrium (Figure 4). It crossed from anterior to posterior, medial to the gallbladder. The transverse colon coursed from right to left, posterior to the superior mesenteric vessels and anterior to the aorta and inferior vena cava (Figure 5). The descending and sigmoid colons were in their usual positions.

The superior mesenteric artery demonstrated normal origin and course. However, the superior mesenteric vein emerged to the left of the artery, crossed anteriorly, and positioned itself to its right. It showed counterclockwise torsion posterior to the pancreas and anterior to the transverse colon.

No dilated small or large bowel loops were observed. There was no bowel wall thickening, no signs of intestinal ischemia, no evidence of acute obstruction, and no peritoneal fluid. Furthermore, there was no hyperdense intraluminal fluid to suggest acute hematoma, nor was there focal hyperdense contrast extravasation to indicate active bleeding at the time of the scan.

**Figure 6:**
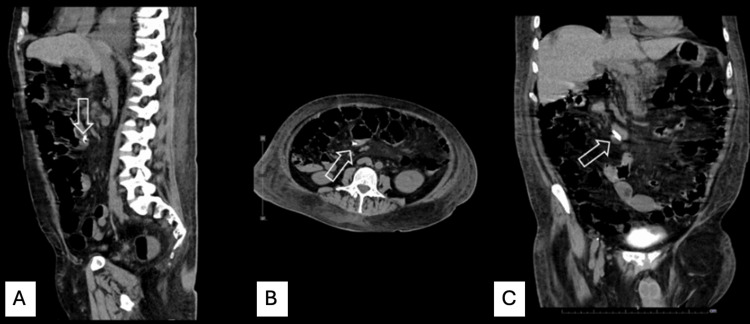
Hemostatic clip Non-contrast CT image (B) and sagittal (A) and coronal (C) reconstructions demonstrating a hyperdense, irregular structure (white arrow) compatible with a hemostatic clip.

Upper gastrointestinal endoscopy performed the following day revealed active bleeding in the duodenum, which was successfully managed with endoscopic placement of a hemostatic clip (Figure 6). Histopathological analysis of biopsies obtained during the procedure demonstrated non-specific chronic inflammatory changes with no evidence of malignancy, alongside a confirmed active *Helicobacter pylori* infection. Because the CT scans had already confirmed an absence of bowel obstruction or ischemia related to the reversed midgut rotation, prophylactic surgical intervention for the anatomical variant was deferred in favor of conservative management.

Following clinical and hemodynamic improvement, with an uncomplicated hospital course and no recurrent bleeding episodes, the patient was discharged on gastroprotective therapy and treatment for *Helicobacter pylori* infection (omeprazole, bismuth, levofloxacin, metronidazole, and amoxicillin) to address the underlying chronic inflammation and prevent recurrent hemorrhage. He remains under close follow-up with internal medicine and gastroenterology. 

## Discussion

During normal embryologic development, between the fifth and tenth weeks of gestation, the midgut undergoes a 270-degree counterclockwise rotation around the superior mesenteric artery. This process results in the normal anatomic configuration of the duodenum, small intestine, and colon [5,7].

Reversed midgut rotation was first described by Landolsi in 1883 [6] and occurs when the physiological rotation is altered, and the intestinal loop rotates clockwise, typically by approximately 90 degrees [2,6]. Consequently, the normal anatomic relationship between the duodenum, transverse colon, and superior mesenteric vessels is reversed. In this variant, the transverse colon is situated posterior to the superior mesenteric artery and superior mesenteric vein, while the duodenum lies anterior to these vascular structures.

Due to the limited number of reported cases, the pathophysiology of this condition is not fully understood. It has been suggested that deficient fixation and increased mobility of the ascending colon and cecum, combined with abnormal anatomic positioning, create a narrow retroarterial space through which the colon passes [4,7]. This predisposes patients to luminal stenosis, transverse colon obstruction, and volvulus -- potentially involving the ileocecal region, right colon, or the entire midgut [3,8].

A review of the literature reveals that the majority of documented adult cases of reversed midgut rotation present with acute mechanical complications. For instance, Singer et al. and Landolsi et al. described cases characterized by severe transverse colon obstruction and cecal volvulus, respectively [1,6]. In those instances, the acute presentations were direct consequences of the anatomical malformation, such as mechanical shearing and compression caused by the narrow retroarterial space.

In stark contrast, our patient completely lacked any signs of mechanical obstruction or vascular compromise, presenting instead with upper gastrointestinal bleeding. This divergence can be logically explained by the distinct etiology of the symptoms. While most symptomatic reversed malrotations in the literature are driven by extrinsic physical torsion of misplaced bowel loops [3,8], our patient's acute presentation was precipitated by an independent intrinsic mucosal pathology.

The reversed midgut rotation in this instance was a clinically silent, incidental finding that was unmasked only because of the exhaustive cross-sectional imaging required to investigate the vague abdominal pain and severe anemia. This distinction holds significant weight for clinical decision-making. Recognizing that a profound anatomical variant can coexist with, but not necessarily cause, an acute gastrointestinal presentation is crucial. As demonstrated in this case, accurately attributing the bleeding to an infectious etiology rather than mechanical torsion allowed the clinical team to avoid an unnecessary exploratory laparotomy and safely pursue conservative, targeted pharmacological management. Differential diagnostic considerations based on the chronic/anatomical presentation before the endoscopic findings in our case included angiodysplasia, superior mesenteric artery syndrome, and intermittent volvulus.

Multiphase CT is the imaging modality of choice for evaluating intestinal malrotation [5,9]. It allows detailed assessment of intestinal and vascular anatomy, which is particularly useful in adults with atypical or non-specific symptoms in the absence of bowel obstruction, as demonstrated in our case. CT also plays a crucial role in surgical planning when intervention is required [4,9].

Characteristic imaging findings of reversed rotation include an ectopic cecum (frequently located in the left upper quadrant), small bowel loops positioned along the midline or within the right hemiabdomen, the transverse colon situated posterior to the superior mesenteric artery, and the duodenum positioned anterior to the mesenteric vessels [2,5,9-11].

## Conclusions

Reversed midgut rotation is an exceedingly rare congenital anomaly that poses a significant diagnostic challenge, particularly when it incidentally coexists with unrelated acute pathologies such as upper gastrointestinal bleeding, rather than presenting with a classic obstructive pattern. Familiarity with its characteristic CT findings, including an ectopic cecum, a retroarterial transverse colon, and an anterior duodenum, is essential to avoid diagnostic delay. This case demonstrates that recognizing these anatomical variants and confirming the absence of mechanical complications allows clinicians to safely attribute acute symptoms to their true intrinsic causes, thereby avoiding unnecessary exploratory surgery and facilitating appropriate conservative management.
